# In vitro comparative study of multimodal imaging nano-assembled microspheres with two clinical drug-eluting beads loaded with doxorubicin

**DOI:** 10.1080/10717544.2023.2197177

**Published:** 2023-04-20

**Authors:** Yiwei He, Tiancheng Xu, Tingting Ding, Yuanchuan Gong, Hui Zeng, Zihan Xi, Yuanxin Ye, Ziyang Song, Ting Pan, Zhewei Zhang, Qian Ma, Lihua Li, Yuqing Zhang, Guoliang Shao

**Affiliations:** a Zhejiang Cancer Hospital, Institute of Basic Medicine and Cancer (IBMC), Chinese Academy of Sciences, Hangzhou, Zhejiang, China; b Second Clinical Medical College of Zhejiang, Chinese Medical University, Hangzhou, China; c School of Automation, Hangzhou Dianzi University, Hangzhou, China

**Keywords:** Comparative study, drug-eluting beads, drug loading, drug release, doxorubicin

## Abstract

DC Beads and CalliSpheres are commonly used microspheres in clinical transcatheter arterial chemoembolization, but these microspheres cannot be visualized by themselves. Therefore, in our previous study, we developed multimodal imaging nano-assembled microspheres (NAMs), which are visualized under CT/MR and the location of embolic microspheres can be determined during postoperative review, facilitating the evaluation of embolic areas and guiding subsequent treatment. Moreover, the NAMs can be carried with positively and negatively charged drugs, increasing the choice of drugs. Systematic comparative analysis of the pharmacokinetics of NAMs with commercially available DC Bead and CalliSpheres microspheres is important for evaluating the clinical application of NAMs. In our study, we compared the similarities and differences between NAMs and two drug-eluting beads (DEBs) in respect to drug loading capacity, drug release profiles, diameter variation and morphological characteristics. The results indicate that NAMs had good drug delivery and release characteristics as well as DC Bead and CalliSpheres in vitro experimental stage. Therefore, NAMs have a good application prospect in transcatheter arterial chemoembolization treatment of hepatocellular carcinoma.

## 1. Introduction

Recently, hepatocellular carcinoma (HCC), due to its increasing incidence, has become the sixth most commonly diagnosed cancer and the third leading cause of cancer death worldwide in 2020 (Garuti et al., 2021; Sung et al., 2021). Due to the concealed onset of liver cancer, most patients are already in the middle to late stage when diagnosed and lose the opportunity of surgery (Granito et al., 2021). Transcatheter arterial chemoembolization (TACE) has been broadly used in unresectable hepatocellular carcinoma by utilizing a microcatheter to hyper-select to the tumor blood supply artery, injecting high concentrations of chemotherapeutic agents and embolizing the tumor nutritional target vessels to increase the local chemotherapeutic effect and cause necrosis of the tumor tissue under the effect of ischemia, hypoxia and local toxicity of chemotherapeutic agents (Lee & Khan, 2017). Commonly used TACE embolization materials include solid embolic agents (such as polyvinyl alcohol particles, gelatin sponge particles, microspheres, etc.) and liquid embolic agents (such as absolute ethanol, iodized oil, etc.). Among them, microspheres have smooth surface, uniform particle size, not easy to aggregate, high targeting to specific tissues and organs, especially drug-eluting beads (DEBs).They physically embolize the tumor’s nutrient vessels, depriving the tumor tissue of nutrients needed for growth, at the same time, the DEBs slowly release chemotherapeutic drugs that can inhibit the synthesis of tumor cell proteins and nucleic acids and cell proliferation, maintaining the local blood concentration of tumor tissue while reducing the damage to normal cells, showing good results in the treatment of malignant tumors (Brown et al., 2016). At present, the commonly used drug-loaded microspheres mainly include DC Bead (BTG, United Kingdom), HepaSphere (Merit Medical, Utah), CalliSpheres (Callisyn Biomedical-Suzhou, Inc.,China) and Tandem (Boston Scientific, Massachusetts). Both DC Bead microspheres and CalliSpheres microspheres are composed of polyvinyl alcohol crosslinked microspheres, and the drug delivery mechanism is the electrostatic interaction between the cations of the drug and the anionic functional groups of the microspheres (Jordan et al., 2010). In the clinic, these traditional DEBs are unable to visualize themselves, and the visualization of the microspheres needs to be assisted by mixing with the contrast agent. However, the exogenous contrast agent will soon be dispersed with the bloodstream and removed from the injection site, and the exact location of the embolized microspheres cannot be confirmed postoperatively, thus affecting the postoperative efficacy evaluation (van Hooy-Corstjens et al., 2008; Sharma et al., 2010; Dreher et al., 2012; Johnson et al., 2016). As a result, imageable drug-loaded microspheres have become a research hot spot and some progress has been made. Currently, the first commercially available radiopaque drug-eluting bead (DC Bead LUMI™) for image guided TACE is in clinic (Levy et al., 2016; Aliberti et al., 2017; Lewis et al., 2018; Mikhail, Pritchard et al., 2022). Imageable microspheres that use conventional and dual energy CT for image-guided-TACE procedure have already been developed (Negussie et al., 2015; Duran et al., 2016; Johnson et al., 2016; Ashrafi et al., 2017; Mikhail et al., 2018; Negussie et al., 2021). These microspheres were loaded with toll-like receptors (TLRs) agonists and immune check point inhibitors (Negussie et al., 2022; Mikhail, Mauda-Havakuk et al., 2022) as well electrostatically layered with antibody (Sakr et al., 2016), enabling DEBs to deliver immunologic and targeted drugs, increasing the choice of local therapeutic agents. And microspheres that containing both a drug and T1 MRI agent and crosslinked with a T2 imaging agent enabled MR imageable (van Elk et al., 2015). Microspheres containing radiopaque gold nanorods and magnetic iron clusters for both computed tomography (CT) and MRI imageable are also being investigated (Kim et al., 2016). In our previous study, we developed multi-modal visible nano-assembled microspheres (NAMs). NAMs can be visualized under CT/MR to identify the position of embolized microspheres during postoperative review, which is conducive to evaluating the embolic area and guiding subsequent treatment. In addition, NAMs can be loaded with positively and negatively charged drugs to increase the choice of drugs (He et al., 2022). Anticancer drugs commonly used in TACE include anthracyclines (doxorubicin, epirubicin and idabicin), platinums (cisplatin and oxaliplatin), fluorouracil and mitomycin. Some clinical studies have shown that doxorubicin-loaded DEBs are more favorable than lipiodol transarterial chemoembolization in terms of hepatotoxicity, adverse effects related to doxorubicin, and patient tolerability (Lammer et al., 2010; Karalli et al., 2019). And doxorubicin is positively charged, which can be loaded by most DEBs and is widely used in D-TACE treatment.

Despite the in vitro characteristics of doxorubicin-loaded DC Bead and CalliSpheres microspheres have been reported (de Baere et al., 2016; Han et al., 2019), there is still a lack of systematic comparative analysis of the pharmacokinetics of DC Bead, CalliSpheres microspheres and NAMs. Therefore, the purpose of this study is to compare the similarities and differences between the NAMs and the two commercially available DEBs in terms of drug loading capacity, drug release profiles, diameter variation and morphological characteristics under the same conditions.

## 2. Materials and methods

### 2.1. Materials

Microspheres evaluated in this study included DC Bead (100–300 μm), Callisphere (100–300 μm). Chloroauric chloride (HAuCl_4_·4H2O) and polyacrylic acid (PAA) were purchased from Sino-pharm Chemical Reagent Co., Ltd. (Shanghai, China). 4-Nitrothiophenol (4-NBT, 90%) was purchased from Fluorochem Ltd. (Derbyshire, United Kingdom). Iron oxide (Fe_3_O_4_) nanoparticles, sodium polystyrene sulfonate (PSS), phosphate buffered saline (PBS), doxorubicin hydrochloride (DOX, 98%) and dopamine hydrochloride (PDA, 98%) were obtained from Aladdin Chemical Reagent Co., Ltd. (Shanghai, China). Embospheres (100–300 μm) were purchased from Merit Medical (United States). Fetalbovine serum (FBS) was purchased from Gibco Life Technologies (United States). Ultrapure water (UPW, 18.2 MΩ) was used for all experiments.

### 2.2. Synthesis of NAMs

Based on our previous study (He et al., 2022), we synthesized the multimodal imageable nano-assembled drug-loaded microspheres, which can simultaneously realize CT/MRI/Raman visualization and loading with positively and negatively charged chemotherapy drugs. Briefly, we first synthesized the multimodal visible nanosphere containing Fe_3_O_4_ nanoparticles, gold nanoparticles, Raman signal molecule 4-NBT and dopamine coating. Then, we assembled the nanospheres with Embospheres to synthesize NAMs after carboxylation modification of the surface of Embosphere, a blank embolization microsphere applied in clinic. Finally, we modified the surface of NAMs with sulfonic acid group to realize the function of loading with doxorubicin.

### 2.3. Doxorubicin loading

Due to clinical application setting, drug additions to DEBs were calculated by one bottle of marketed microspheres. Therefore, the amounts of doxorubicin added and loaded in this study were based on one package of marketed DEBs, even though some differences in amount might exist between the products from each brand. One package of marketed DC Bead is 2 mL, Callisphere is 1 mL, and one package NAMs is based on one package of marketed Embosphere, which is 1 mL. DEBs were rinsed by the ultrapure water for three times, discarding the liquid supernatant. Take 1 bottle of marketed microspheres and later adding doxorubicin (100 mg) solution at a concentration of 12.5 mg/mL or 25 mg/mL, based on commonly used clinical drug delivery concentrations (Shao et al., 2021). The mixture was incubated by gentle rotation at room temperature. The concentration of the supernatant was quantified at 0, 15, 30, 60, 90 min, and 24 h. The concentration of the doxorubicin supernatant was quantified using a high performance liquid chromatography-mass spectrometry (HPLC-MS) system (Thermo Fisher UltiMate 3000). The chromatographic column used was a Hypersil Gold C18 (2.1 mm × 100 mm, 1.9 μm) from Thermo Scientific. The loading capacity was calculated as follows: Loading capacity = initial doxorubicin amounts − the sum of residual doxorubicin amount in the supernatant at the various time points.

### 2.4. Doxorubicin release

For the evaluation of elution, 1 package of drug-eluting beads (the specification of DC Bead is 2 mL, and the specification of CalliSpheres and NAMs is 1 mL) loaded with 100 mg doxorubicin at a concentration of 25 mg/mL was placed in 250 mL of PBS at pH = 5.6, PBS at pH = 7.4, PBS + 10% FBS at pH = 5.6 and PBS + 10% FBS at pH = 7.4 separately, to investigate the influence of FBS and pH. And drug released by rotation at 37 °C. Three replicates were evaluated and samples were collected at 15, 30 min, 1, 2, 4, 6, 8, 12, 18, 24, 48, 72, 96 and 120 h, then replenish the solvent to 250 mL. FBS-containing samples were first treated with liquid–liquid extraction (Yuan et al., 2020; Alshabrawy et al., 2021). The concentration of doxorubicin was determined by the HPLC-MS method as described earlier. The release amount at each time point was calculated as follows: Release amount = *C_n_* × *V* − *C_n_*_– 1_×residual volume. And the cumulative release amount = the sum of the release amount of each time point.

### 2.5. Morphology and physical properties

The optical microscope and a Zeiss Sigma 300 scanning electron microscope (SEM, Zeiss, Germany) were used to obtain the morphological properties of each kind of drug-eluting beads (DEBs). One milliliter of microspheres was sampled, and at least 100 batches were counted each time to obtain the average diameters. The diameters before and after drug loading were evaluated using dynamic light scattering method by the LS 13 320 Particle Size Analyzer (Beckman Coulter Co.), and the shrinkage percentage was calculated as follows:

Percentage of shrinkage (%)=(diameters of unloaded DEBs−diameters of DEBs after loading or elution)/diameters of unloaded DEBs×100%

### 2.6. Statistics analysis

Data were primarily presented as mean ± standard deviation. SPSS Statistics 23.0 and GraphPad Prism 8.0 were used for statistical analysis and plotting. Comparison between two groups was determined by *t* test, and paired t test was used for pre–post comparison of the same group. The difference was statistically significant with *p* < .001.

## 3. Results and discussion

### 3.1. Doxorubicin loading

**Figure 1.**
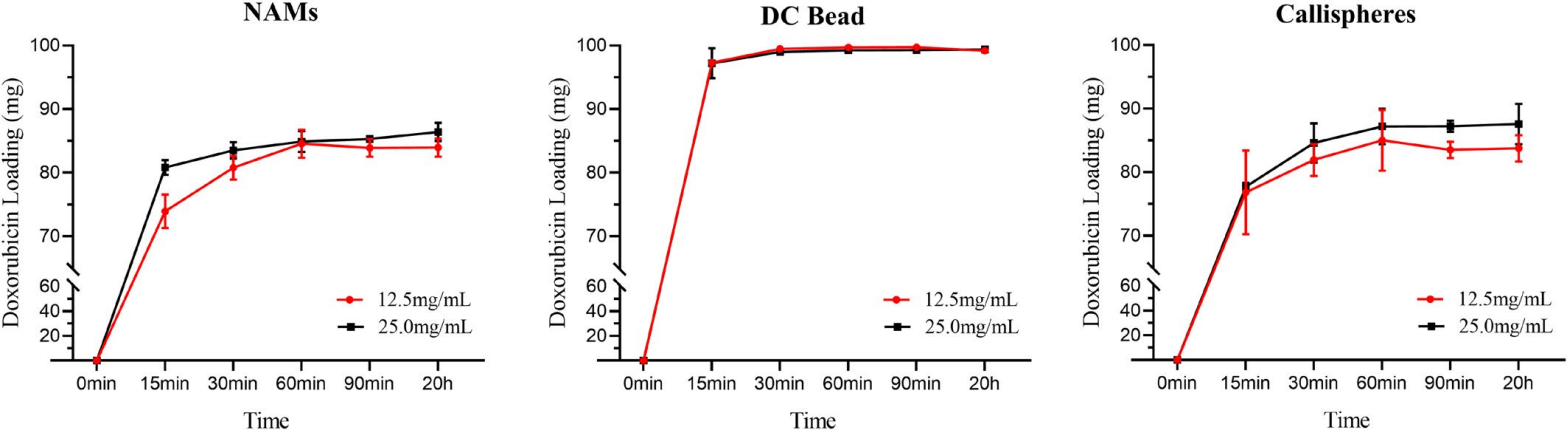
Doxorubicin loading amount of NAMs, DC Bead and Callispheres at the drug concentration of 12.5 mg/mL or 25 mg/mL. Error bars indicate standard deviations (*n* = 3).

The drug loading amount of three types of DEBs for doxorubicin are shown in Figure 1 and Supplementary Figure S1. In general, all the three types of microspheres could quickly load doxorubicin. The drug loading amount of DC Bead could reach plateau level within 15 min, while the drug loading amount of NAMs and Callispheres increased rapidly in the first 15 min and remained slowly increasing after 15 min. At the same amount of doxorubicin to be added, loading amount and loading efficiency of doxorubicin were positively correlated with drug concentration. At the drug concentration of 12.5 mg/mL, the drug loading amount of DC bead in 15 min was 97.31 ± 0.46 mg, which was higher than that of Callispheres (76.83 ± 6.58 mg) and NAMs (73.93 ± 2.64 mg), and at the drug concentration of 25 mg/mL, the drug loading amount of DC bead in 15 min was 97.21 ± 3.36 mg, which was higher than NAMs (80.82 ± 1.15 mg) and Callispheres (77.72 ± 0.60 mg). It can be seen that the drug loading capacity of DC bead is basically the same at the above two concentrations, and the drug loading capacity of DC Bead is better than that of the other two kinds of microspheres. There was no significant difference in the drug loading capacity of NAMs and Callispheres.

The drug-loading capacity of the three DEBs varies, and the loading amount and loading efficiency of all three DEBs were acceptable considering clinical application dosage. The doxorubicin loading capacity of the three types of DEBs was different, which might be related to two factors. The first reason is probably related to drug loading mechanism. The common drug loading mechanism of DEBs is electrostatic interaction, based on negative charges on the microspheres and positive charges in doxorubicin, such as Callispheres and DC beads (Ashrafi et al., 2017; Zhang et al., 2017). While NAMs utilized covalent bond binding for the drug loading. The sulfonate group on the NAMs surface was used to bind doxorubicin specifically. Another possible reason is that, due to the clinical application settings, we compare the microspheres according to a commercially available specification, but there are some differences in the volume, quality and quantity of the packaging for each brand of microspheres.

### 3.2. Doxorubicin release

**Figure 2.**
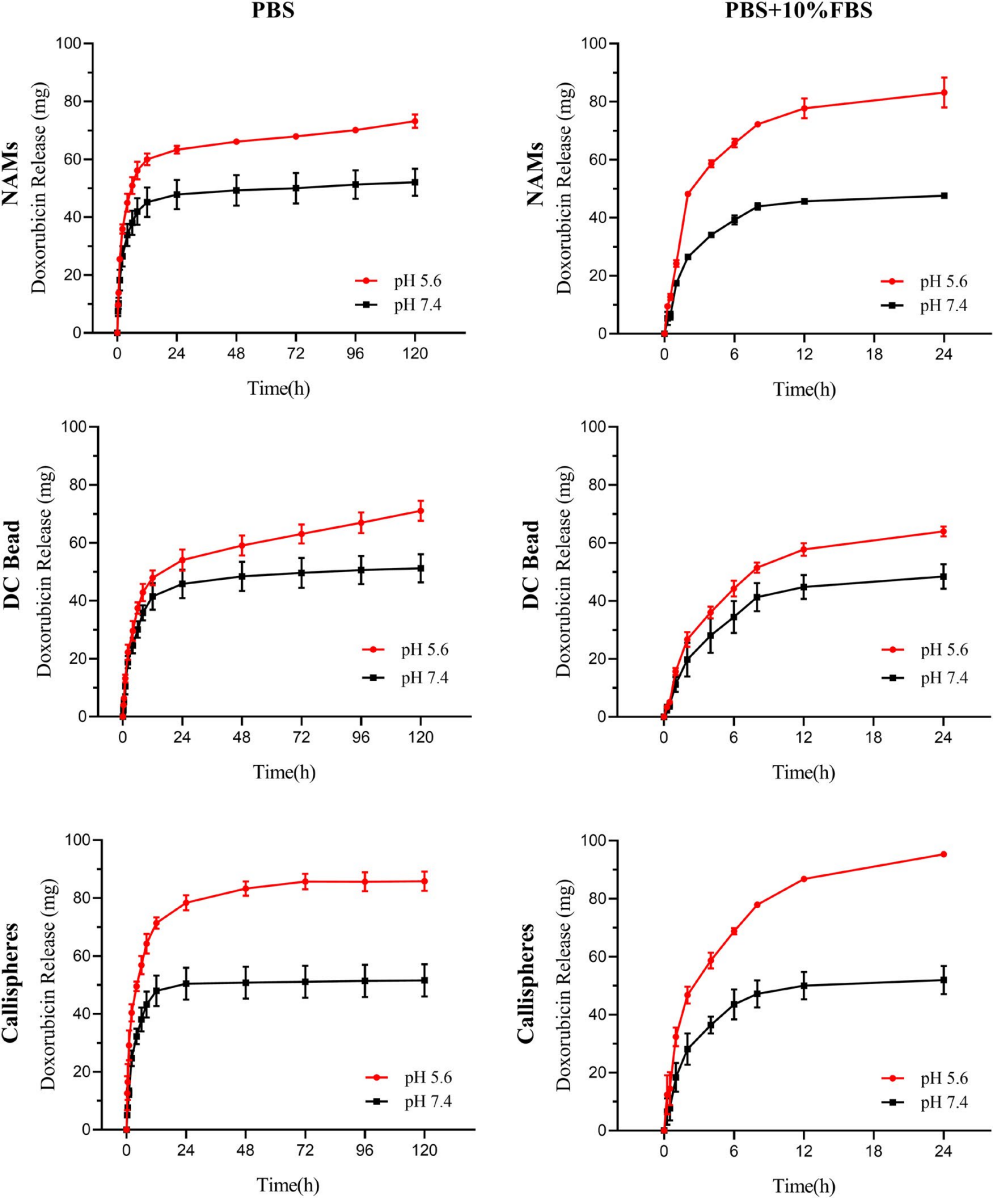
Doxorubicin release amount of NAMs, DC Bead and Callispheres in PBS with or without 10% FBS at pH = 5.6 or pH = 7.4. Error bars indicate standard deviations. (*n* = 3).

The results of doxorubicin cumulative release amount are shown in Figure 2. In general, all the three types of DEBs first released rapidly and reached a plateau at the same time (around 12 h), then slowly sustained released. In PBS at pH = 5.6, the cumulative release amount of NAMs, DC Bead and Callispheres were 73.19 ± 2.33 mg, 71.07 ± 3.46 mg and 85.84 ± 3.35 mg within 120 h, respectively. While in PBS at pH = 7.4, the cumulative release amount of NAMs, DC Bead and Callispheres were 52.08 ± 4.71 mg, 51.20 ± 4.85 mg and 51.60 ± 5.60 mg within 120 h, respectively. It follows that doxorubicin cumulative release amount of Callispheres was slightly higher than that of NAMs and DC Bead within 120 h in PBS at pH = 5.6, while there was no significant difference in doxorubicin cumulative release amount of three types DEBs in PBS at pH = 7.4. And compared with neutral environment, microspheres released more drugs in weakly acidic environment. And the microenvironment of tumor tissue is exactly weak acidic environment, which is favorable for the release of microspheres drugs (Su et al., 2022). Since FBS is susceptible to bacterial denaturation, which affected the measurement of drug concentration, we only measured the data for the first 24 h in the PBS + 10% FBS group. Under the condition of 10% FBS addition concentration, FBS could further accelerate drug release in the first 24 h, and the results were similar to a previous study (Lu et al., 2021). In that study, the authors investigated CalliSpheres, DC Bead and HepaSphere loaded with raltitrexed for release in PBS and PBS + 10%FBS, and the results showed FBS could further accelerate the release of raltitrexed. This mechanism needs to be further investigated. However Chen et al. showed that the maximum doxorubicin release percentage of CalliSpheres in saline with 20% FBS group was decreased compared with the saline group (Chen et al., 2021). This indicated that when the concentration of FBS is too high, the excess protein adsorbed on the surface of microspheres would limit the release of drug.

### 3.3. Morphology and physical properties

**Figure 3.**
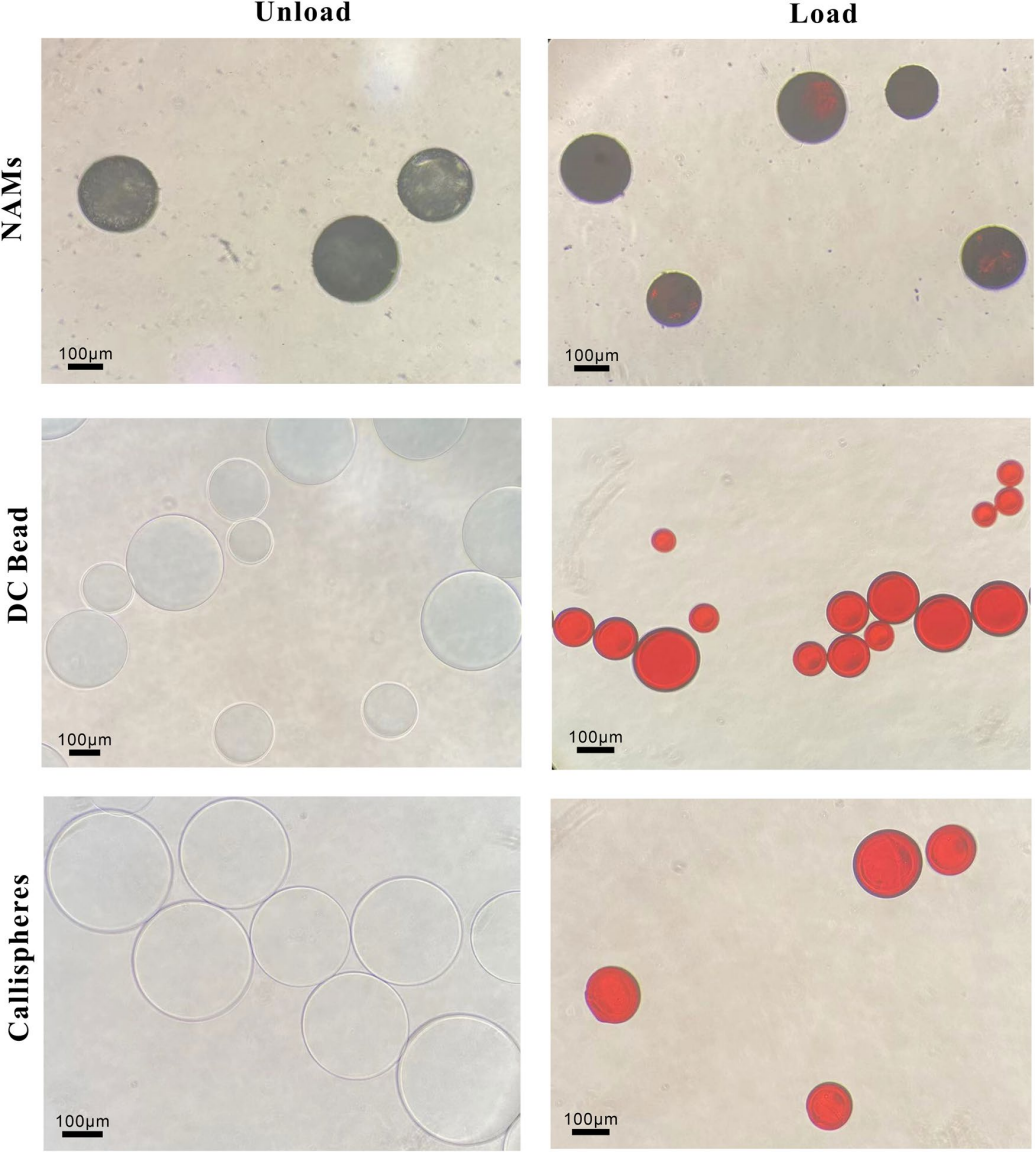
Morphologies of unloaded and doxorubicin-loaded of NAMs, DC Bead and Callispheres microspheres under optical microscope. The scale bar is 100 μm.

**Figure 4.**
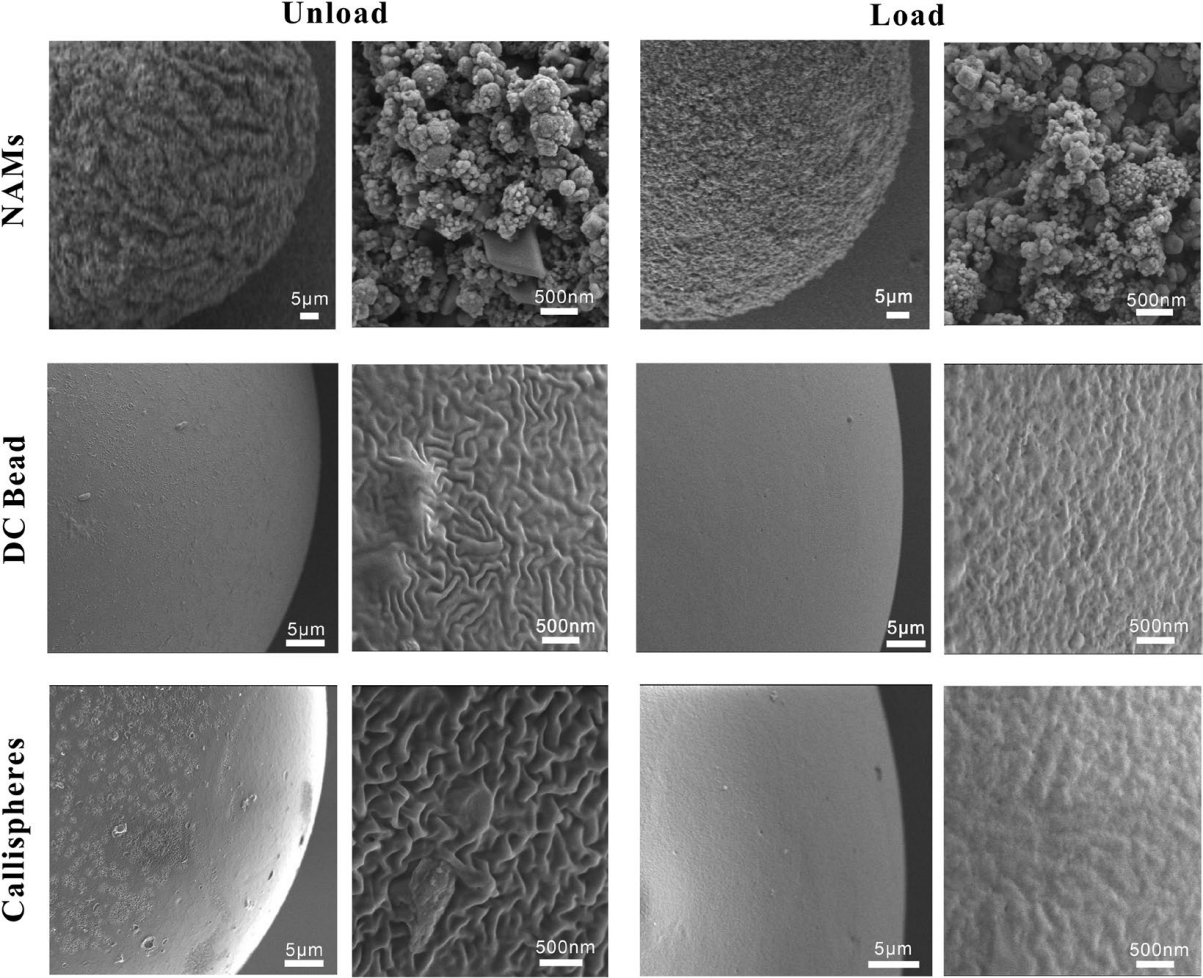
SEM images of NAMs, DC Bead and Callispheres microspheres before/after doxorubicin loading. The scale bar is 5 μm for the left and 500 nm for the right.

**Figure 5.**
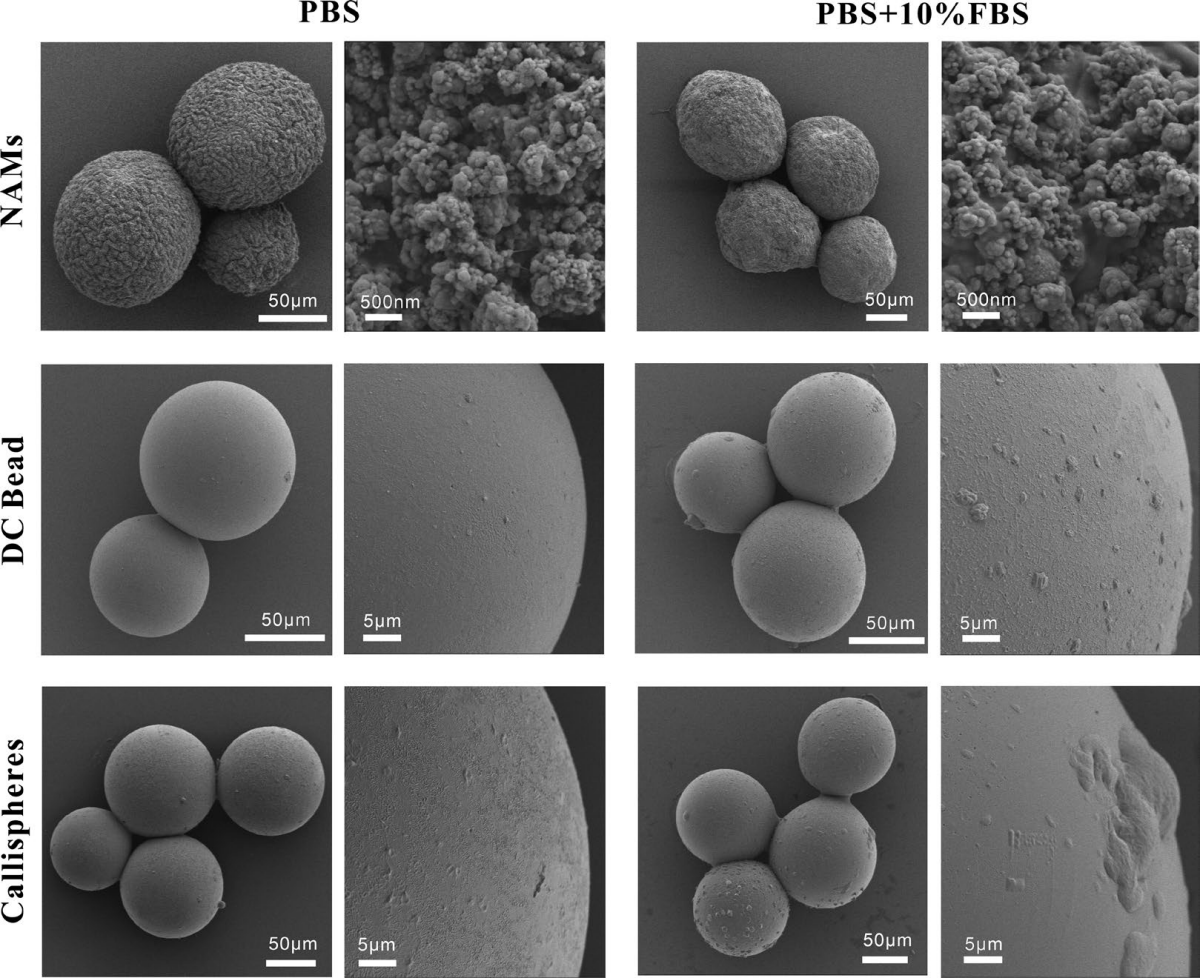
SEM images of postelution of NAMs, DC Bead and Callispheres microspheres in PBS with or without 10% FBS at pH = 5.6. The left scale bar is 50 μm, the right scale bar for NAMs is 500 nm, and for DC Bead and Callispheres is 5 μm.

The results of the optical microscope are given in Figure 3. We can see that NAMs were black before drug loading due to the multimodal nano-visible spheres assembled on its surface, and after doxorubicin loading, they showed deep red color. DC Bead and Callispheres were both transparent blue spheres, and after drug loading, they appeared bright red. SEM images before and after doxorubicin loading are shown in Figure 4, in which all three types of microspheres exhibit uneven surface before drug loading and flattened surface after drug loading. This may be because small drug molecules stick to the surface of the microspheres, so the surface looks smoother. As can be seen from Figure 5, the surface of NAMs with the release solvent containing FBS were smoother, which may be probably due to the adsorption of proteins on the surface of the microspheres filling the gaps on the surface. DC Bead and Callispheres, compared with the released solvent as simple PBS, the surface of microspheres with released solvent containing FBS were visible as round and flat particles, which might be proteins adsorbed on the surface.

**Figure 6.**
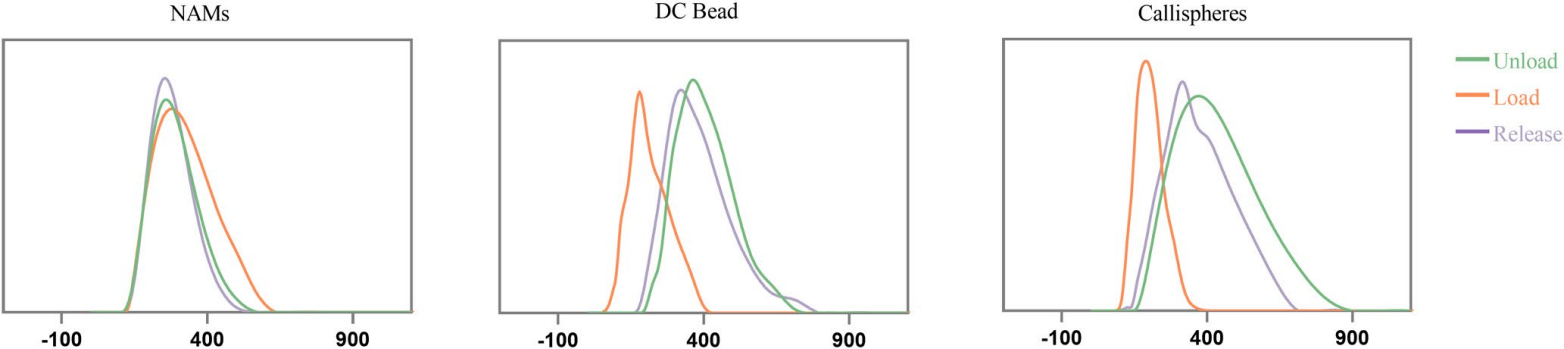
Diameter changes of NAMs, DC Bead and Callispheres microspheres before/after doxorubicin loading and release.

Subsequently, dynamic light scattering method was used to determine the diameters of unloaded, doxorubicin-loaded and postelution of NAMs, DC Bead and Callispheres (Figure 6). And the results showed that the diameter before loading were 253.23 ± 0.71 μm, 333.76 ± 4.84 μm and 331.35 ± 4.85 μm respectively. After loading, the diameters were 274.56 ± 4.15 μm (*p* = .01), 176.30 ± 3.48 μm (*p* < .001) and 183.44 ± 1.03 μm (*p* < .001) respectively (Table 1). The shrinkage percentages were –8.42%, 47.18% and 44.64% for NAMs, DC Bead and Callispheres, respectively. While after elution, the diameters were determined as 259.84 ± 11.72 μm, 317.40 ± 4.60 μm and 307.83 ± 3.09 μm, respectively, which demonstrated a recovery in sizes from shrinkage to within 10% of the original ranges (4.9% and 7.1% for DC Bead and Callispheres, respectively). The size of NAMs did not change significantly after drug loading and release (Table 1).

**Table 1.** Diameters of unloaded, doxorubicin-loaded and postelution of NAMs, DC Bead and Callispheres.

	Diameters(μm)						
DEBs type	Unloaded	Doxorubicin-loaded	*p*	Diameter shrinkage ratio	Diameters (μm) after drug release	*p*	Diameter shrinkage ratio
NAMs	253.23 ± 0.71	274.56 ± 4.15	.01	–8.42%	259.84 ± 11.72	.416	–2.61%
DC Bead	333.76 ± 4.84	176.30 ± 3.48	< .001	47.18%	317.40 ± 4.60	.0084	4.90%
Callispheres	331.35 ± 4.85	183.44 ± 1.03	< .001	44.64%	307.83 ± 3.09	.0280	7.10%

## 4. Conclusions

In conclusion, the present study identified the similarities and differences of multimodal imaging NAMs and two commercially available microspheres loaded with doxorubicin. NAMs had good drug delivery and release characteristics as well as DC Bead and CalliSpheres in vitro experimental stage. Moreover, combining the results of our previous study (He et al., 2022), NAMs can be visualized under CT and MR and load with negatively charged chemotherapy drug compared to DC Bead and Callispheres, having a good application prospect in TACE treatment of hepatocellular carcinoma.

## Supplementary Material

Supplemental Material

## Data Availability

The authors confirm that the data supporting the findings of this study are available within the article [and/or] its supplementary materials.

## References

[CIT0001] Aliberti C, Carandina R, Sarti D, et al. (2017). Transarterial chemoembolization with DC Bead LUMI™ radiopaque beads for primary liver cancer treatment: preliminary experience. Future Oncol 13:1–8. 10.2217/fon-2017-0364 29063780

[CIT0002] Alshabrawy AK, Bergamin A, Sharma DK, et al. (2021). LC-MS/MS analysis of vitamin D(3) metabolites in human serum using a salting-out based liquid-liquid extraction and DAPTAD derivatization. J Chromatogr B Analyt Technol Biomed Life Sci 1173:122654. 10.1016/j.jchromb.2021.122654 33819798

[CIT0003] Ashrafi K, Tang Y, Britton H, et al. (2017). Characterization of a novel intrinsically radiopaque drug-eluting bead for image-guided therapy: DC Bead LUMI^TM^. J Control Release 250:36–47. 28188808 10.1016/j.jconrel.2017.02.001 PMC5416940

[CIT0004] Brown KT, Do RK, Gonen M, et al. (2016). Randomized trial of hepatic artery embolization for hepatocellular carcinoma using doxorubicin-eluting microspheres compared with embolization with microspheres alone. J Clin Oncol 34:2046–53. 26834067 10.1200/JCO.2015.64.0821 PMC4966514

[CIT0005] Chen Q, Shu L, Sun Y, et al. (2021). In vitro drug loading, releasing profiles, and in vivo embolic efficacy and safety evaluation of a novel drug-eluting microsphere (CalliSpheres). Cancer Biother Radiopharm. doi:10.1089/cbr.2020.3766. 33493417

[CIT0006] de Baere T, Plotkin S, Yu R, et al. (2016). An in vitro evaluation of four types of drug-eluting microspheres loaded with doxorubicin. J Vasc Interv Radiol 27:1425–31. 27402527 10.1016/j.jvir.2016.05.015

[CIT0007] Dreher MR, Sharma KV, Woods DL, et al. (2012). Radiopaque drug-eluting beads for transcatheter embolotherapy: experimental study of drug penetration and coverage in swine. J Vasc Interv Radiol 23:257–64.e4. 22178039 10.1016/j.jvir.2011.10.019 PMC3360470

[CIT0008] Duran R, Sharma K, Dreher MR, et al. (2016). A novel inherently radiopaque bead for transarterial embolization to treat liver cancer – a pre-clinical study. Theranostics 6:28–39. 26722371 10.7150/thno.13137 PMC4679352

[CIT0009] Garuti F, Neri A, Avanzato F, et al. (2021). The changing scenario of hepatocellular carcinoma in Italy: an update. Liver Int 41:585–97. 33219585 10.1111/liv.14735

[CIT0010] Granito A, Facciorusso A, Sacco R, et al. (2021). TRANS-TACE: prognostic role of the transient hypertransaminasemia after conventional chemoembolization for hepatocellular carcinoma. J Pers Med 11:1041. 34683182 10.3390/jpm11101041 PMC8539564

[CIT0011] Han X, Chen Q, Sun Y, et al. (2019). Morphology, loadability, and releasing profiles of callispheres microspheres in delivering oxaliplatin: an in vitro study. Technol Cancer Res Treat 18:1533033819877989. 31630671 10.1177/1533033819877989 PMC6801889

[CIT0012] He Y, Zhang Y, Gong Y, et al. (2022). Multimodal imaging of nano-assembled microspheres loaded with doxorubicin and Cisplatin for liver tumor therapy. Front Bioeng Biotechnol 10:1024174. 36213082 10.3389/fbioe.2022.1024174 PMC9539659

[CIT0013] Johnson CG, Tang Y, Beck A, et al. (2016). Preparation of radiopaque drug-eluting beads for transcatheter chemoembolization. J Vasc Interv Radiol 27:117–26.e3. 26549370 10.1016/j.jvir.2015.09.011 PMC6663477

[CIT0014] Jordan O, Denys A, De Baere T, et al. (2010). Comparative study of chemoembolization loadable beads: in vitro drug release and physical properties of DC bead and hepasphere loaded with doxorubicin and irinotecan. J Vasc Interv Radiol 21:1084–90. 20610183 10.1016/j.jvir.2010.02.042

[CIT0015] Karalli A, Teiler J, Haji M, et al. (2019). Comparison of lipiodol infusion and drug-eluting beads transarterial chemoembolization of hepatocellular carcinoma in a real-life setting. Scand J Gastroenterol 54:905–12. 31287338 10.1080/00365521.2019.1632925

[CIT0016] Kim DH, Li W, Chen J, et al. (2016). Multimodal imaging of nanocomposite microspheres for transcatheter intra-arterial drug delivery to liver tumors. Sci Rep 6:29653. 27405824 10.1038/srep29653 PMC4942792

[CIT0017] Lammer J, Malagari K, Vogl T, et al. (2010). Prospective randomized study of doxorubicin-eluting-bead embolization in the treatment of hepatocellular carcinoma: results of the PRECISION V study. Cardiovasc Intervent Radiol 33:41–52. 19908093 10.1007/s00270-009-9711-7 PMC2816794

[CIT0018] Lee EW, Khan S. (2017). Recent advances in transarterial embolotherapies in the treatment of hepatocellular carcinoma. Clin Mol Hepatol 23:265–72. Dec 29113030 10.3350/cmh.2017.0111 PMC5759999

[CIT0019] Levy EB, Krishnasamy VP, Lewis AL, et al. (2016). First human experience with directly image-able iodinated embolization microbeads. Cardiovasc Intervent Radiol 39:1177–86. 27206503 10.1007/s00270-016-1364-8 PMC7831151

[CIT0020] Lewis AL, Willis SL, Dreher MR, et al. (2018). Bench-to-clinic development of imageable drug-eluting embolization beads: finding the balance. Future Oncol 14:2741–60. 29944007 10.2217/fon-2018-0196 PMC6219436

[CIT0021] Lu E, Tie J, Liu L, et al. (2021). An in vitro comparative study of three drug-eluting beads loaded with raltitrexed. Cancer Biother Radiopharm. doi:10.1089/cbr.2021.0251. 34767737

[CIT0022] Mikhail AS, Mauda-Havakuk M, Negussie AH, et al. (2022). Evaluation of immune-modulating drugs for use in drug-eluting microsphere transarterial embolization. Int J Pharm 616:121466. 35065205 10.1016/j.ijpharm.2022.121466 PMC9139086

[CIT0023] Mikhail AS, Pritchard WF, Negussie AH, et al. (2018). Mapping drug dose distribution on CT images following transarterial chemoembolization with radiopaque drug-eluting beads in a rabbit tumor model. Radiology 289:396–404. 30106347 10.1148/radiol.2018172571 PMC6219695

[CIT0024] Mikhail AS, Pritchard WF, Negussie AH, et al. (2022). Cone-beam computed tomography-based spatial prediction of drug dose after transarterial chemoembolization using radiopaque drug-eluting beads in woodchuck hepatocellular carcinoma. Invest Radiol 57:495–501. 35239613 10.1097/RLI.0000000000000864 PMC11493181

[CIT0025] Negussie AH, de Ruiter QMB, Britton H, et al. (2021). Synthesis, characterization, and imaging of radiopaque bismuth beads for image-guided transarterial embolization. Sci Rep 11:533. 33436734 10.1038/s41598-020-79900-z PMC7804415

[CIT0026] Negussie AH, Dreher MR, Johnson CG, et al. (2015). Synthesis and characterization of image-able polyvinyl alcohol microspheres for image-guided chemoembolization. J Mater Sci Mater Med 26:198. 26105830 10.1007/s10856-015-5530-3 PMC6663481

[CIT0027] Negussie AH, Mikhail AS, Owen JW, et al. (2022). In vitro characterization of immune modulating drug-eluting immunobeads towards transarterial embolization in cancer. Sci Rep 12:21886. 36535979 10.1038/s41598-022-26094-1 PMC9763333

[CIT0028] Sakr OS, Berndt S, Carpentier G, et al. (2016). Arming embolic beads with anti-VEGF antibodies and controlling their release using LbL technology. J Controlled Release 224:199–207. 10.1016/j.jconrel.2016.01.010 26780173

[CIT0029] Shao G, Zou Y, Lucatelli P, et al. (2021). Chinese expert consensus on technical recommendations for the standard operation of drug-eluting beads for transvascular embolization. Ann Transl Med 9:714. 33987412 10.21037/atm-21-1678 PMC8106009

[CIT0030] Sharma KV, Dreher MR, Tang Y, et al. (2010). Development of “imageable” beads for transcatheter embolotherapy. J Vasc Interv Radiol 21:865–76. 20494290 10.1016/j.jvir.2010.02.031 PMC2876341

[CIT0031] Su T, Huang S, Zhang Y, et al. (2022). miR-7/TGF-β2 axis sustains acidic tumor microenvironment-induced lung cancer metastasis. Acta Pharm Sin B 12:821–37. 35251919 10.1016/j.apsb.2021.06.009 PMC8896986

[CIT0032] Sung H, Ferlay J, Siegel RL, et al. (2021). Global Cancer Statistics 2020: GLOBOCAN estimates of incidence and mortality worldwide for 36 cancers in 185 countries. CA Cancer J Clin 71:209–49. 33538338 10.3322/caac.21660

[CIT0033] van Elk M, Ozbakir B, Barten-Rijbroek AD, et al. (2015). Alginate microspheres containing temperature sensitive liposomes (TSL) for MR-guided embolization and triggered release of doxorubicin. PloS One 10:e0141626. 26561370 10.1371/journal.pone.0141626 PMC4641710

[CIT0034] van Hooy-Corstjens CS, Saralidze K, Knetsch ML, et al. (2008). New intrinsically radiopaque hydrophilic microspheres for embolization: synthesis and characterization. Biomacromolecules 9:84–90. 18067259 10.1021/bm7008334

[CIT0035] Yuan TF, Le J, Wang ST, Li Y. (2020). An LC/MS/MS method for analyzing the steroid metabolome with high accuracy and from small serum samples. J Lipid Res 61:580–6. 31964762 10.1194/jlr.D119000591 PMC7112139

[CIT0036] Zhang S, Huang C, Li Z, et al. (2017). Comparison of pharmacokinetics and drug release in tissues after transarterial chemoembolization with doxorubicin using diverse lipiodol emulsions and CalliSpheres Beads in rabbit livers. Drug Deliv. 24:1011–17. 28660787 10.1080/10717544.2017.1344336 PMC8241087

