# Supplementary material for: In vitro comparative study of multimodal imaging nano-assembled microspheres with two clinical drug-eluting beads loaded with doxorubicin
Source: Drug Deliv. 2023 Apr 20;30(1):2197177. doi: 10.1080/10717544.2023.2197177 (PMC10120570; doi:10.1080/10717544.2023.2197177)
Supplement: Supplemental Material [file IDRD_A_2197177_SM7341.docx]

Supplementary Material


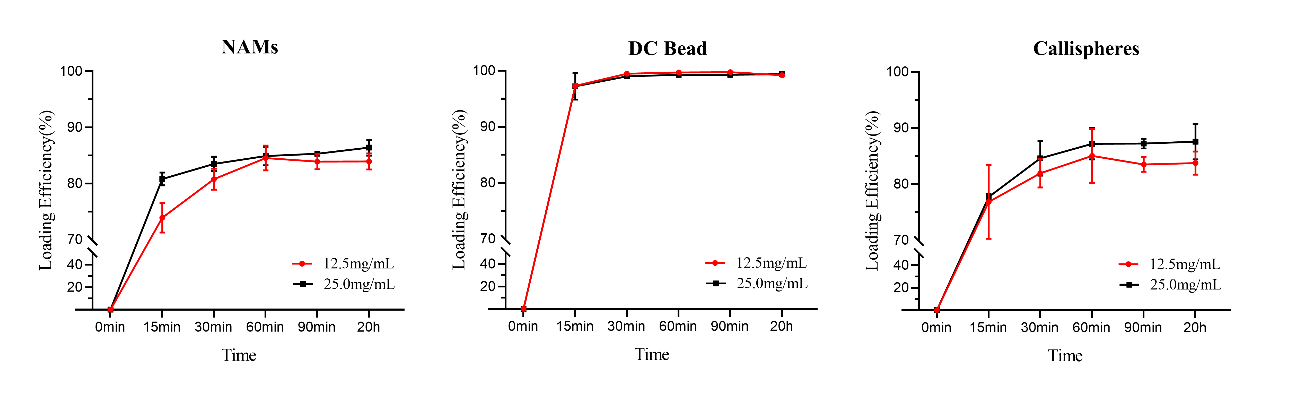


**Supplementary Figure 1.** Doxorubicin loading efficiency of NAMs, DC Bead and Callispheres at the drug concentration of 12.5mg/ml or 25 mg/ml. Error bars indicate standard deviations (n=3).
